# Temperature- and species-specific infection could modify stream insect communities

**DOI:** 10.1007/s00284-026-04897-z

**Published:** 2026-05-07

**Authors:** Sarah A. Taig, Galen Holt, Georgia K. Dwyer, Rebecca E. Lester

**Affiliations:** Deakin University, Locked Bag 20000, Geelong, VIC 3220 Australia

## Abstract

To maintain diverse communities under changing environmental conditions, species must be able to concentrate density feedback within species compared to between species. Density feedback can occur via multiple mechanisms, including predator partitioning, and is often mediated by the physical environment. If species respond differently to the environment (e.g. susceptibility to predators changes under different conditions) then environmental fluctuations can provide the necessary separation of density feedback to promote coexistence. Additionally, directional change in environmental conditions (as with climate change) can yield overall shifts in community composition. We assess possible pathways that a fungal-like parasite (*Saprolegnia* spp.) could modify these effects in a community of Hydrobiosidae caddisflies. We exposed caddisfly egg masses from four species to four controlled temperature regimes in the laboratory and determined mortality from infection. *Saprolegnia* spp. infection in caddisfly egg masses is species specific, a signature for predator partitioning which can influence coexistence. The caddisfly species also differ in how temperature alters their infection rates, with dramatic shifts in relative infection rates depending on the duration and timing of periods of high temperature. Our findings suggest that *Saprolegnia* spp. infection could limit dominant species, shift community composition, and help stabilise coexistence under fluctuating temperatures. While we show key conditions of coexistence are met, quantifying the effect of these temperature-mediated infection rates would require measuring the extent to which they modify density-dependent feedback.

**Supplementary Information:**

The online version contains supplementary material available at 10.1007/s00284-026-04897-z.

## Introduction

The earth supports myriad diverse communities with species coexisting in shared habitats with relationships that range from mutually beneficial to detrimental for one or all involved. Different species relationships include predation, parasitism, pollination, and many more [1]. The environment in which these species reside can play an important role in how they relate to one another [1, 2]. Species relationships can be shaped by resource availability, climate, and habitat structure [3]. These interactions between species relationships and the environment can often lead to shifts in population dynamics and subsequently community structure [4]. To maintain diversity in communities, competing organisms in the same area require density-dependent feedbacks within a species to be stronger than those between species. This condition is most easily met when species have similar average fitnesses and low niche overlap [2, 5]. Stabilising methods and equalising methods can make these conditions of coexistence more likely [2].

Shifting environmental conditions can alter relationships by strengthening one species’ fitness and weakening another species from the same area, particularly if the latter is a strong competitor [6]. If the more competitive species is weakened specifically due to its higher density, then shifts in population can stabilise coexistence if the impacts shift as species densities change, such as with frequency-dependent predation [7–9]. In contrast, if reduced performance is due to physiological differences between species, these are tied to species identity and so would be equalising [7–9]. Natural events such as disease outbreaks, floods or heatwaves are mechanisms which can induce responses that alter species densities and performance [10]. With disease, host specialisation can be equalising or stabilising depending on whether infection rates are higher for the competitively dominant species due to differing host susceptibility or through its abundance (frequency-dependent selection). For example, in a community of two competing bacteria (*Pseudomonas fluorescens* and *P. aeruginosa*), the introduction of a bacteriophage reduced the density of the numerically dominant bacterium, which changed depending on the environmental temperature. Rather than infecting both species equally, this frequency-dependent partitioning altered competitive interactions, leading to more numerically even coexistence [11].

There are many emergent diseases, and these new host-pathogen relationships can alter the performance of species and their interactions. Moreover, host-pathogen relationships can be altered by a number of environmental factors including water quality, pH [12], resource availability, humidity, and temperature [13, 14]. Impacts from new and changing host-pathogen relationships can alter diversity maintenance (as described above through differing species fitness), so it is important to understand the implications of these shifts for community structure and diversity maintenance. For example, host-pathogen relationships are frequently mediated by temperature, causing changes to both overall infection rates and shifts in relative infection rates between species [15]. Increased temperatures can lead to both increased infection rates (e.g. wheat species have higher rates of infection of wheat streak mosaic virus at increased temperatures; [16]) and decreased infection rates (e.g. a herbaceous plant has lower infection rates of anther-smut disease at high temperatures; [17]). If infection is increased and population densities are decreased, then populations may lose genetic diversity and become more susceptible to other disturbances [18]. Temperature increases could also alter relative mortality outcomes in similar species, leading to changes in density-dependent interactions and shifts in diversity [15].

If the relationship between temperature and infection is different for different species, then density-dependent relationships within the community will shift. Shifts in mean temperatures in this case would most affect the most susceptible species, altering average fitness differences and relative abundances [19]. If competitively dominant species are most susceptible, the resulting equalisation can make coexistence more likely, while increasing susceptibility of weaker species would make stable coexistence less likely. Temperature fluctuations could provide opportunities for stabilising coexistence [20]. Stabilising coexistence can occur through temperature fluctuations if different species are favoured by lower infection rates at different times or locations, depending on the temperature. Such variation in performance can allow low density species to escape density dependence generated by their competitors when the low-density species have high performance in different areas or times. This separation is captured by the storage effect and fitness-density covariance coexistence mechanisms [2, 21].

**Fig. 1:**
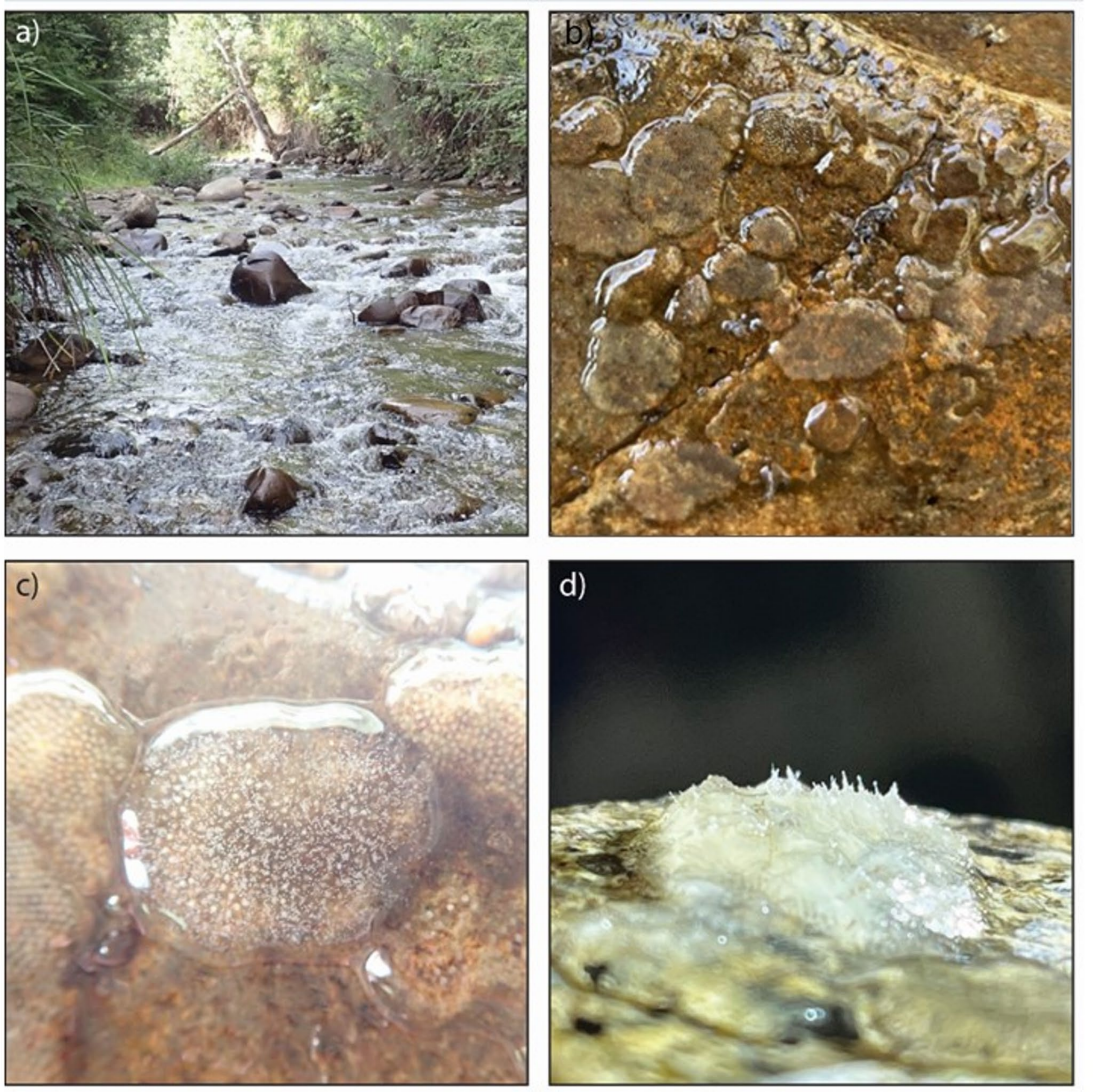
Examples of **(a)** rocks within riffles, **(b)** egg masses in a cluster on a rock, **(c)** a *U. rubiconum* egg mass which is infected, and **(d)** an *E. turbidum* egg mass which is infected. Egg mass size differs between species, ranging from 5–25 mm [28]. Evidence of infection in panels c) and d) is cloudiness or white spots in the spumaline as well as a changed shape of the spumaline all of which is caused by hyphae spreading throughout the egg mass. Photo credits: S. Taig

In this study, we investigate species- and temperature-dependent *Saprolegnia* spp. infection in Hydrobiosidae caddisfly eggs. The focal species, *Ulmerochorema rubiconum* [22, 23], *U. seonum* [24], *Taschorema evansi* [24] and *Ethochorema turbidum* [22], are caddisflies (Trichoptera) with four life stages (egg, larva, pupa and adult). Female hydrobiosid caddisflies lay a single clutch of eggs (in a gelatinous egg mass) in their lifetimes on the underside of emergent rocks in rivers and streams [25] (Fig. 1). Hydrobiosids have varying oviposition habits, with some preferring fast flows over slow flows and vice versa [26, 27]. Each egg mass can be identified to the species level by their shape and number of eggs [28] although the eggs within vary little in size [29]. The eggs are covered in spumaline, a gelatinous substance which holds the eggs in place and may provide some protection against high water flows and predators [30]. The spumaline for *U. rubiconum* and *U. seonum* is thicker compared to *T. evansi* and *E. turbidum*. These four species lay their egg masses in the greatest numbers in the summer, when the weather is warm and dry, and the number of egg masses is reduced in the colder months [31]. Egg masses occur as single masses or in clusters of multiple touching egg masses that often contain multiple species [32]. Mortality due to *Saprolegnia* spp. infection had a significant increase in the Australian summer of 2019/2020 in caddisfly egg masses in the rivers surrounding Marysville, Australia [33]. Infection can be identified as the egg masses become cloudy and have a fungal-like appearance along with the eggs turning white as they die [33] (Fig. 1).

Within one caddisfly community, infection rates of *Saprolegnia* spp. vary among species and cause mortality. The probability of infection and consequent mortality of the most abundant species *U. rubiconum* increases with high water temperature for periods of 12 h or more, which were initiated the day after the egg massed were laid [34]. It is possible that similar temperature dependence will occur in other species that are observed to suffer infection in this natural system, such as *U. seonum*,* T. evansi* and *E. turbidum*. Additionally, as temperature causes complex physiological changes in development [35], the timing of temperature increases during development may alter infection probability. Infection may increase at a lower rate when temperatures are increased later in the egg period, due to greater development of eggs and their immune system [36]. Further, eggs exposed to different environmental conditions may have greater defence against infection which could lead to increased variation in the onset and spread of infection. If temperature-dependent infection in each species occurs differently, leading to different mortality consequences, it could shift relative performance of the different species, potentially altering both equalisation and stabilisation of coexistence in this community.

**Fig. 2:**
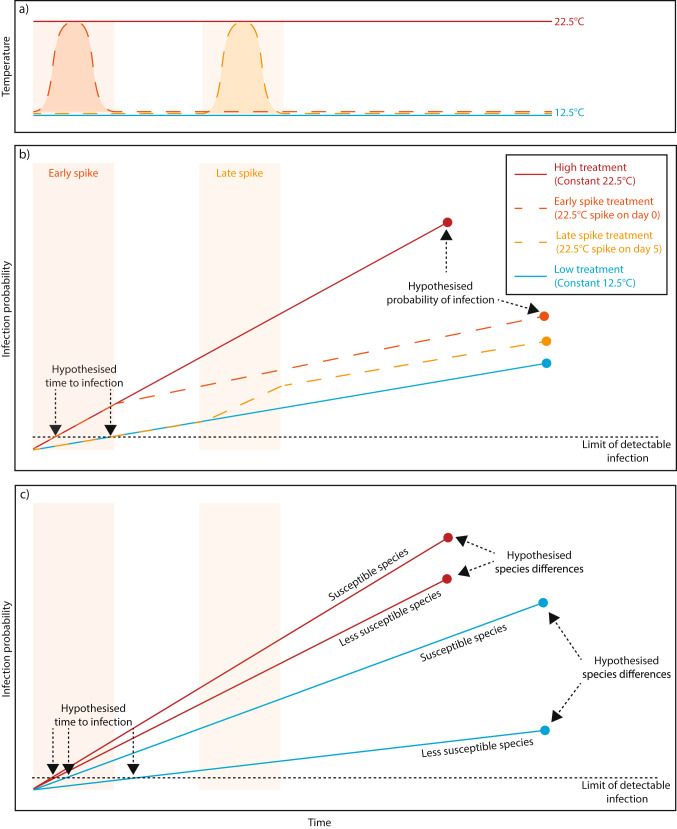
Illustrations of potential species- and temperature-specific responses to infection. **(a)** The temperature regimes to which egg masses from different treatments were exposed. The High and Low treatments were constant at 22.5 °C and 12.5 °C, respectively. The spike treatments increased from 12.5 °C to 22.5 °C for 12 h during the experiment, before returning to 12.5 °C, which occurred on the first day for the Early spike and the fifth day for the Late spike treatment. **(b)** We hypothesised that the day of the first detectable infection (Q1; dotted black line) and infection probability (Q2) would be influenced by the temperature treatments. We expected that detectable infection would occur earlier in the High and Early spike treatments than the Late spike and Low treatments. We also hypothesised that realised infection probability (endpoint dots) would increase with temperature, likely as a consequence of a higher rate of infection per day (slopes of lines). Additionally, infection probability may increase at a lower rate when temperatures were increased later in the egg period, due to greater development of eggs, leading to the greatest infection probability in the High treatment, followed by the Early spike, then Late spike and finally the Low treatment. **(c)** We hypothesised that, across the temperature patterns (Low and High treatments portrayed), there would be species that are more susceptible to infection leading to greater infection probability and fewer days until first detectable infection than species that are less susceptible. We expected that detectable infection would occur earlier for the more susceptible than the less susceptible species. The difference between more and less susceptible species in each of these characteristics may also differ between treatments. As illustrated here, the less-susceptible species is more sensitive to temperature relative to the more-susceptible species and so receives a much greater relative fitness boost in cooler temperatures

Clearly, different temperature responses by the host and parasite can combine to yield complex infection dynamics. We quantify differences in caddisfly mortality from *Saprolegnia* spp. infection in four hydrobiosid species exposed to temperature patterns differing in the duration and onset timing of increased temperatures (Fig. 2a). We answer the following questions:

Q1. Does the day of infection vary with different temperature patterns and caddisfly species?

### Hypothesis 1

The day of infection will be earlier for some species (assuming species vary in susceptibility) and eggs exposed to higher temperatures.

Q2. Does the probability of infection, and therefore mortality, vary with different temperature patterns and caddisfly species?

### Hypothesis 2

Infection probability will increase with earlier and longer exposure to increased temperatures. Species will differ in infection probability and the response of infection to temperature.

We use these questions to identify any species- or temperature-specific difference in infection characteristics. In identifying these infection characteristics, we aimed to determine whether *Saprolegnia* spp. infection in caddisflies has characteristics necessary to influence coexistence through changing relative populations of each life stage or overall populations if the egg stage is limiting (Fig. 2b and c illustrate patterns suggesting a contribution to coexistence). In particular, we aim to identify whether species’ infection susceptibilities are differently sensitive to environmental conditions. From our experiment, some possible implications for coexistence mechanisms are: (1) if species are differently susceptible to infection on average, this would indicate shifts in average fitness and potentially equalisation and frequency-dependent partitioning; (2) if infection is temperature dependent, it would indicate a shift in mortality rates across the community under different climates; and (3) if infection is differently temperature-dependent among species, it would indicate shifts in dominance under different climates and the potential for stabilisation with temperature fluctuations.

## Methods

### Experimental Methods

In February (summer) 2023, rocks with egg masses present on them (all laid in a single night) were collected the morning after laying from the Steavenson and Taggerty Rivers. Further sample collection methods can be found in Appendix S1 Sect.  1. Egg masses of the species *U. rubiconum*,* U. seonum*,* E. turbidum* and *T. evansi* were collected on 43 rocks and placed into individual experimental containers filled with river water before being transported to the Deakin University laboratory (Waurn Ponds, Victoria). Each rock was randomly allocated to one of four temperature treatments (representing a range of observed field temperatures from 2017), including: two short-term heatwaves, defined as exposure to increased temperatures for part of the experiment, with either early and late onset timings (Early spike, 0–12 h and Late spike, 96–108 h, respectively); a long duration heatwave which had a consistently high temperature (High); and a control which had consistently low temperature (Low; Fig. 2a). The High and Low treatments remained at 22.5 °C (common summer/autumn maximum temperature) and 12.5 °C (common summer minimum temperature), respectively, for the duration of the experiment (34 days; the number of days required for all egg masses to either become fully infected or to fully hatch). The spike treatments were exposed to 12-hour temperature increases to 22.5 °C and remained at 12.5 °C for the period before and after the temperature spike. To maintain the desired temperatures, the containers holding each rock were stored within large constant temperature chambers (A1000, Conviron, Pembina, North Dakota, USA). To maintain dissolved oxygen levels and replicate water movement, aerators were fitted to each container. Two chambers were set at each temperature (22.5 °C and 12.5 °C) and rocks were repeatedly randomised across suitable chambers at the beginning and end of each spike treatment (i.e. days 0, 1, 5 and 6; Appendix S1 Sect.  2). Rocks assigned to spike treatments were moved from low to high temperature chambers at the start time of their assigned spike, and back to the low temperature 12 h later. This movement around chambers ensured that individual chambers or locations within chambers did not alter hatching or infection characteristics within the treatment groups.

Additional water collected at the time the egg masses were collected was split across four containers and kept at temperatures consistent with each of the treatment groups. Water from these containers was added to experimental containers to their original level if water levels dropped from evaporation. The volume of water added to experimental containers was recorded.

Infected egg masses collected the previous day in the field were blended in 1 L of river water and 20 ml of the solution was added to each of the rock containers and the additional water containers. The infected solution was added to increase exposure to *Saprolegnia* spp. spores and hyphae to ensure infection occurred during the experiment. An additional experiment which did not include the infected solution as a mechanism to increase infection can be found in Appendix S1 Sect.  3.

Random allocation of rocks to treatments and an odd number of total rocks sampled resulted in uneven sample sizes across treatments (Low = 10, Early spike = 11, Late spike = 11, High = 11). Variation in the number of egg masses on each rock and of each species contributed further differences among treatments (Table 1a). To reliably identify each individual egg mass throughout the experiment, and to note whether egg masses were touching other egg masses on a rock, maps were created for each rock uniquely labelling each egg mass.

The eggs within egg masses were monitored daily, and the percentages of infection and hatching were recorded as well as the number of dead eggs which were not obviously related to infection. This monitoring continued until every individual egg within an egg mass on a rock became infected or hatched, which occurred in 7 to 34 days.

**Table 1:** **(a)** The number of egg masses of each species in each treatment group included in the experiment. **(b)** The egg masses in species and treatments included in the analysis for day of infection and coefficient of variation for day of infection (Q1). These include only egg masses which had mortality from infection recorded. The species *U. seonum* was excluded due to low infection. (c) The eggs in species and treatments included in the analysis of infection probability (Q2). These include every egg from the experimental egg masses. (d) The egg masses in species and treatments included in the analysis of the coefficient of variation for infection (Q2). The high treatment *U. seonum* group was not included as a coefficient of variation could not be determined as there was only one egg mass

	*U. rubiconum *	*U. seonum*	*E. turbidum *	*T. evansi*	Treatment total
(a) *Total egg masses in species and treatments*					
Low	26	7	8	4	45
Early Spike	46	30	5	5	86
Late Spike	26	30	4	5	65
High	43	1	7	6	57
Species total	141	68	24	20	253
(b) *Total egg masses in species and treatments for day of infection analyses*					
Low	22	NA	4	2	28
Early Spike	36	NA	3	3	42
Late Spike	21	NA	1	4	26
High	40	NA	7	5	52
Species total	119	NA	15	14	148
(c) *Total eggs in species and treatments for infection probability analyses*					
Low	5044	721	9384	1840	16 989
Early Spike	8924	3090	5865	2300	20 179
Late Spike	5044	3090	4692	2300	15 126
High	8342	103	8211	2760	19 416
Species total	27 354	7004	28 152	9200	71 710
(d*) Total egg masses in species and treatments for coefficient of variation of maximum infection*					
Low	26	7	8	4	45
Early Spike	46	30	5	5	86
Late Spike	26	30	4	5	65
High	43	NA	7	6	56
Species total	141	67	24	20	252

### Data Analysis

We conduct Poisson (Q1), or logistic beta-binomial (Q2) regressions to test our hypotheses. Further, we use Wald Type I and Type II chi-squared tests on analyses of deviance (non-gaussian version of an analysis of variance) to assess the overall statistical significance of the treatment term in the models. As eggs are found within an egg mass, in a cluster, and on a rock, identifiers of each of these levels (egg mass, cluster and rock) were included as nested random effects to account for common correlations within these groups. The number of eggs within each egg mass was determined by the mean number of eggs for each species found by Lancaster and Glaister [28]: *U. rubiconum* = 194, *E. turbidum* = 1173, *T. evansi* = 460 and *U. seonum* = 103. In some analyses, there are species with a smaller number of egg masses per treatment and therefore we use a conservative approach to interpret these results by focusing on the overall patterns between groupings rather than specific effect sizes. Data analysis used the open-source software R [37, 38] using packages glmmTMB [39], spaMM [40] and emmeans [41].

To determine how the day of first infection was altered in different species and treatments and whether there was any interaction between the two terms (Q1), we used a Poisson regression, where the response variable was the day on which infection was first recorded for an egg mass, and rock and cluster identity were included as random effects. This analysis excluded egg masses that never became infected (i.e. we excluded those where no mortality was seen by infection) as they did not have a first day of infection which could be included (i.e. the response was undefined). The results presented are conservative as the exclusion of uninfected egg masses censors the data in such a way that differences between treatments may be missed. The species *U. seonum* was also excluded from this analysis due to low rates of infection. The total number of egg masses included was 148 (see Table 1b for a summary of egg mass numbers in each species and treatment). A type II analysis of deviance was performed on the complete model to determine which terms explained a significant amount of deviance within the model.

To determine how the probability of infection, and therefore mortality, from *Saprolegnia* spp. varied with different temperature patterns and different caddisfly species or an interaction between the two (Q2), we used a logistic (beta binomial) regression. These are standard logistic regressions which include beta-distributed random effects [42]. The response variable was the infection status of each egg, determined by the maximum recorded infection of an egg mass throughout the experiment. The maximum recorded infection was used to determine infection probability as infection can move through egg masses and fade once it has run its course. Maximum recorded infection is therefore a conservative estimate of total infection as it is not always possible to determine where infection has occurred previously if the eggs have disintegrated within the mass, and it is not possible to determine whether missing eggs are due to infection or hatching. The total number of eggs included in this analysis was 71,710 eggs (see Table 1c for a summary of egg mass numbers in each species and treatment). An analysis of deviance was performed on the complete model to determine which terms explained a significant amount of deviance within the model. Due to the complexity of the model a type I analysis of deviance was necessary in which it is possible for the order of the terms to change the significance of the results. Therefore, to identify any effect of the term order we also present results from an analysis of deviance where the terms were reordered.

In analysing the first day of infection (Q1) and infection probability (Q2), differences in variation among species and treatments were apparent. Consequently, we conducted a *post-hoc* estimation of the coefficient of variation (CV) and standard error for both the first day of infection and infection probability. As CV is calculated over all rocks in each treatment and species group, the quality of estimates varies with sample size, and so we calculate standard error by dividing the CV by the square root of two times the number of egg masses. This approach was intended to visualize differences between groups in an exploratory manner to suggest future research rather than as part of the primary analysis. The number of egg masses used in the CV analyses included all of those in the day to infection analyses (Table 1b) and all but one in the infection probability analyses (Table 1d). The missing egg mass is a single *U. seonum* from the high treatment where the CV was unable to be determined.

The methods and analyses were repeated in an experiment using only *U. rubiconum* egg masses to support some of the findings (Appendix S1 Sect.  3).

## Results

Q1. Does the day of infection vary with different temperature patterns and caddisfly species?

**Fig. 3:**
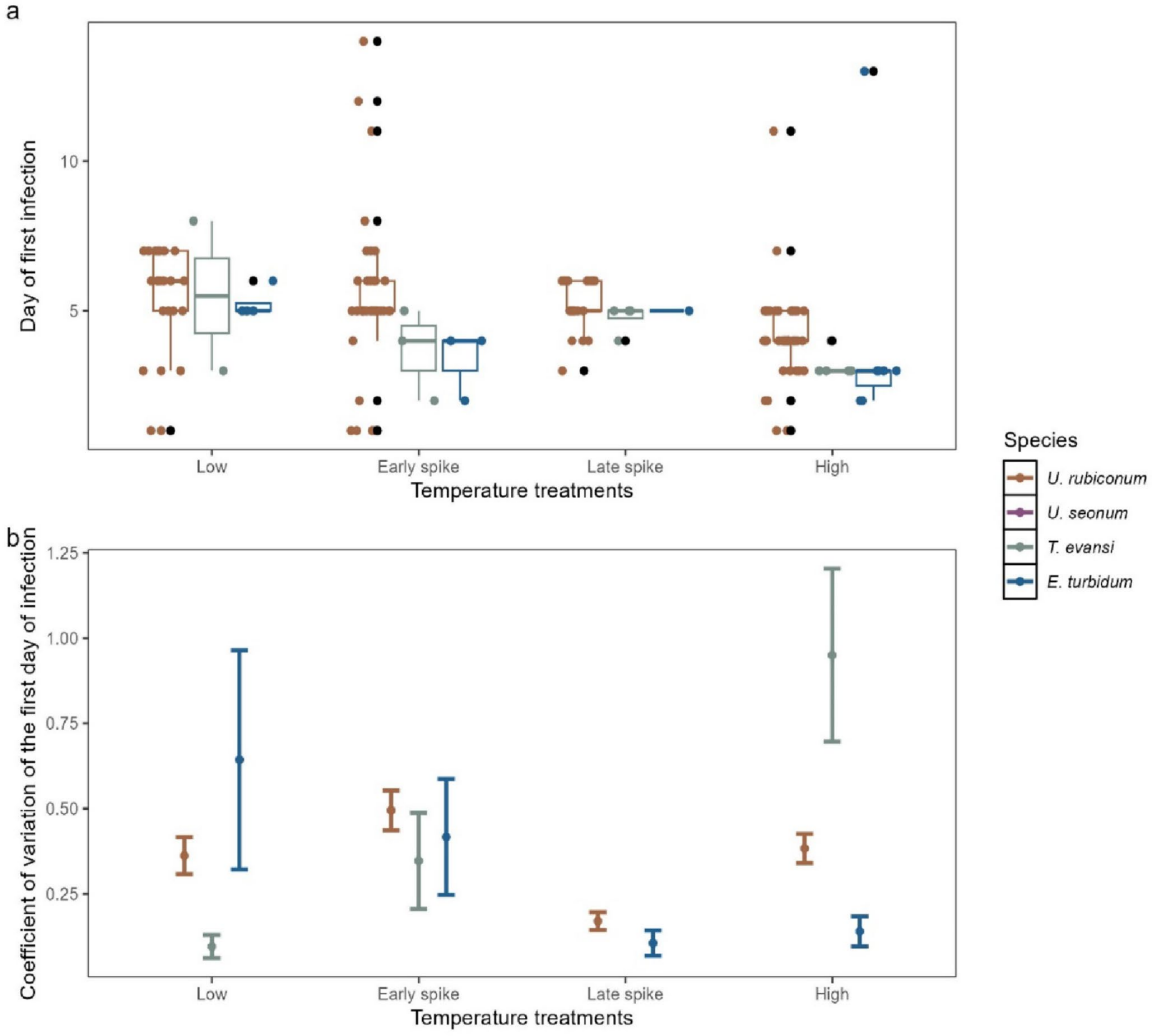
**(a)** Day of first visible infection by species and treatment. **(b)** Coefficient of variation for the day of first visible infection. CV is calculated over all rocks in a treatment x species combination and so is represented here as its value (point) and the SE of that estimate, accounting for sample size (error bars). In panel (a) the coloured points each represent one egg mass. The bold line within the boxplots indicates the median for each grouping. The boxes represent the interquartile ranges. Whiskers extend to the smallest and largest values within 1.5 times the interquartile ranges and black points correspond to outlying egg masses. For panel (b) the coloured points represent the coefficient of variation for each species and the error bars are the standard error. For panels (a) and (b) 148 egg masses were included in these analyses

For the first day of infection, we found no significant differences between species, treatments or their interaction (Fig. 3a; Table 2a; Appendix S1 Sect.  4). The species *U. seonum* was excluded from this analysis due to low rates of infection causing limited ability to determine the mean first day of infection for each treatment. Since the interaction was not significant, a model without the interaction was also run and indicated no significant terms (Appendix S1 Sect.  5). These results match those of the single-species experiment (*U. rubiconum* only) where the treatment term was not significant (Appendix S1 Sect.  3).

**Table 2:** **(a)** Analysis of deviance table (Type II Wald chi squared tests) for the first day of infection. **(b)** Analysis of deviance table (Type I Wald chi squared tests) for the probability of infection

	Chi squared	Degrees of freedom	p-value
**(a) First day of infection**			
Treatment	5.46	3	0.141
Species	2.17	2	0.338
Treatment : Species	2.97	6	0.812
**(b) Probability of infection**			
Treatment	10.8	3	0.0128
Species	65.2	3	< 0.0001
Treatment : Species	36.9	9	< 0.0001

While there was no difference among species or treatments in the of day of infection, there were differences in the CV for this variable between species in each of the treatments (Fig. 3b). The species which had the largest CV varied depending on the treatment. For example, in the Low treatment, *E. turbidum* had the largest CV and *T. evansi* the lowest, while the pattern was reversed in the High treatment. The Spike treatments had smaller differences between species with error bars overlapping in most cases. The species *T. evansi* was not included in the Late spike treatment as only one egg mass was suitable in this treatment for analysis. The species *U. seonum* was excluded from this analysis due to low rates of infection and so limited ability to estimate variation in day of infection.

Q2. Does the probability of infection, and therefore mortality, vary with different temperature patterns and caddisfly species?

**Fig. 4:**
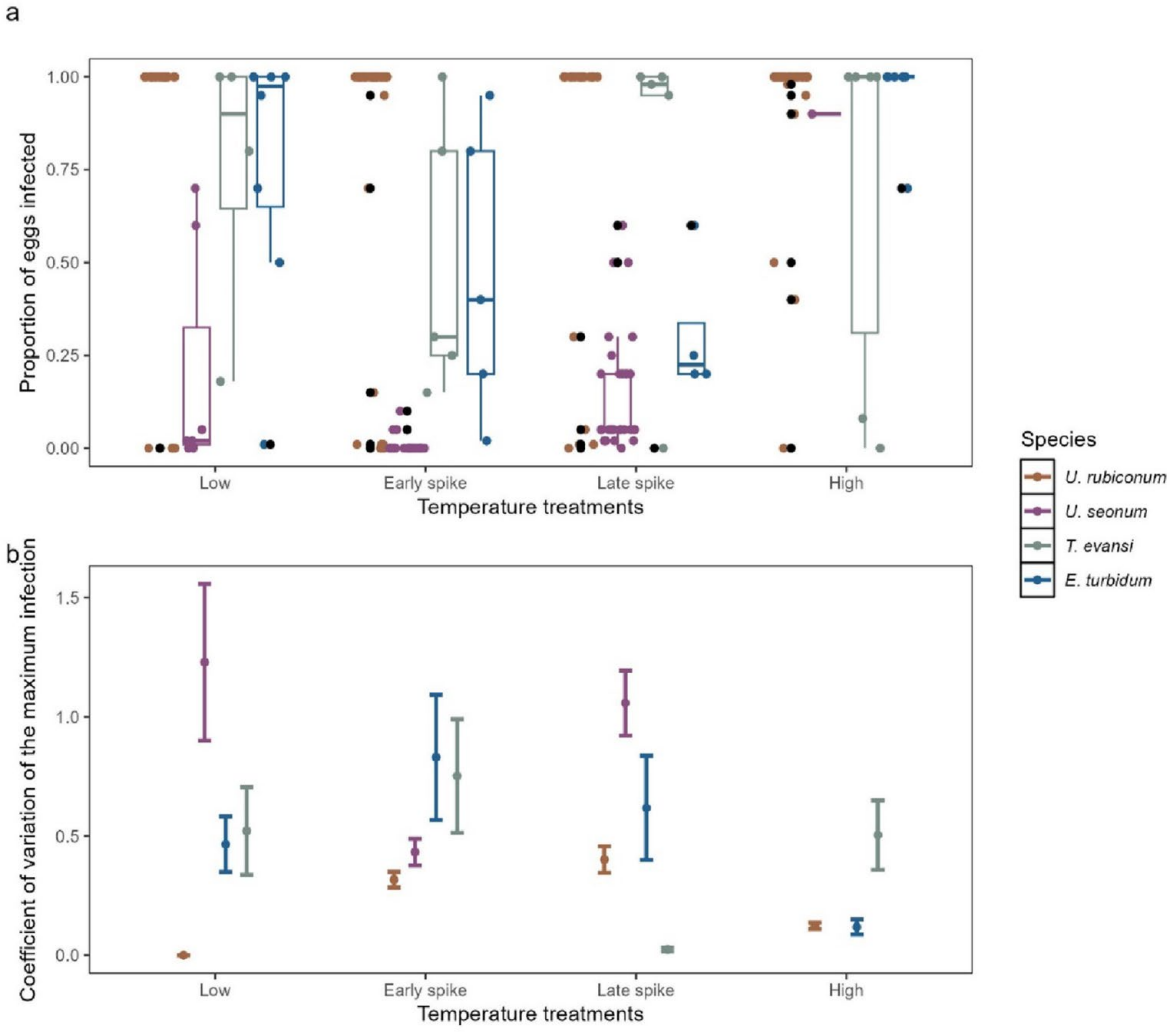
**(a)** Proportion of eggs infected with *Saprolegnia* across species and treatments. Different proportions of eggs were infected in egg masses of each species in response to the different temperature patterns. *U. rubiconum* (brown) eggs became largely infected in each of the treatments whereas *T. evansi* (grey) and *E. turbidum* (blue) infection rates differed in each treatment in different ways. Lastly, *U. seonum* (purple) eggs had small amounts of infection. **(b)** The coefficient of variation for the maximum infection. CV is calculated over all rocks in a treatment x species combination and so is represented here as its value (point) and the SE of that estimate, accounting for sample size (error bars). In panels (a) the coloured points each represent one egg mass. The bold line within the boxplots indicates the median for each grouping. The boxes represent the interquartile ranges. Whiskers extend to the smallest and largest values within 1.5 times the interquartile ranges and black points correspond to outlying egg masses. For panel (b) the coloured points represent the coefficient of variation for each species and the error bars are the standard error. For panel a) 71 710eggs from all the egg masses (253) were included in panel and all egg masses except one (252) were included in panel b). The missing egg mass in panel d) is a single *U. seonum* from the high treatment where the coefficient of variation was unable to be determined

The probability of infection varied with species, treatment and their interaction, based on an analysis of deviance of the complete model (Table 2b; Fig. 4a). Results varied slightly from an analysis of deviance where the order of the terms were altered, where only the species and interaction terms were significant (Appendix S1 Sect.  6). The complete model with estimates of each term is given in Appendix S1 Sect.  7. Using the Low treatment *U. rubiconum* eggs for comparison as this is the dominant species, specific significant differences exist with *U. seonum* (Estimate = -11.2, standard error = 4.2, *p* = 0.0091) and with Late spike / *T. evansi* (Estimate = -10.2, standard error = 4.7, *p* = 0.031) and High / *E. turbidum* (Estimate = 10.1, standard error = 4.3, *p* = 0.018). Additionally, the impact of treatments varied, including in rank order of infection proportion, among species (Fig. 4a; Appendix S1 Sect.  8). Further support that the different treatments lead to different infection probabilities is seen in the single-species experiment (*U. rubiconum* only; Appendix S1 Sect.  3), where the Treatment term was significant in the analysis of deviance and the High treatment had significantly greater infection probability than the Low treatment in the complete model.

There were differences in the CV for maximum infection between species in each of the treatments (Fig. 4b, Appendix S1 Sect.  3). For example, *U. rubiconum* and *E. turbidum* had lower coefficients of variation in the Low and High treatments compared to the spike treatments. The other two species and the single species experiment did not follow this pattern. The species with the highest and lowest CV varied among treatments. *Ulmerochorema seonum* was not included in the High treatment due to only one suitable egg mass being included in this treatment for this analysis. In general, the High treatment had a smaller CV relative to other treatments. Those other treatments overall had similar coefficients of variation.

## Discussion

Infection characteristics varied in complex ways among host species and with different temperature patterns. There were differences in infection probabilities among the four caddisfly host species observed in this study which changed with differing temperature patterns. In each of the treatments, *U. seonum* eggs had the lowest infection probability and *U. rubiconum* eggs had the greatest infection probability, though relative infection rates changed across species and treatments. Infection rates in *T. evansi* eggs and *E. turbidum* changed rank-order depending on the temperature pattern to which they were exposed. There were also differences between species in each of the treatments in the CV for maximum infection. The onset of infection was not different between species or treatments but there were species differences in the CV for day of first infection of egg masses within the treatments. These differences in infection have characteristics suggestive of both stabilising and equalising coexistence mechanisms.

The differences in infection probability between species seen here are suggestive of predators as a mechanism for stable coexistence within the environment [7]. Most striking is that the numerically dominant species, *U. rubiconum*, has consistently highest infection rates from *Saprolegnia* spp. These infection rates may contribute to equalising mechanisms if infection of *U. rubiconum* is consistently high, due for example to physiological differences, and so reduces its overall competitive dominance. It is also possible that these infections are stabilising if they are highest in *U. rubiconum* simply because it is numerically dominant and would switch to other species if they became dominant. Without testing the system across changes in abundance we are unable to separate these two effects but, in either case, increased infection in the most-abundant species makes persistence of the others more likely.

Infection probability is also mediated by changes in temperature, where increased temperatures for the duration of the egg period led to higher rates of infection. Though species vary, the significant main effect of treatment indicates an overall shift to higher infection rates, particularly in the High treatment. The result of this overall increased infection could be reductions in population densities if later life stages do not compensate for increased egg mortality [34, 43] This highlights that natural temperature changes, particularly increases in heatwaves and average temperatures as predicted with climate change, could exacerbate infection risk, possibly decreasing caddisfly populations. A decrease in population densities would leave populations more susceptible to other dangers, for example, due to low genetic diversity which can cause further declines in populations from inbreeding and mutation accumulation [18].

Species- and temperature-specific infection probabilities together show patterns consistent with an ability to support coexistence by shifting relative fitness outcomes for species depending on the environment. Infection rates were highest for all species in the High treatment but, in the Low and both spike treatments, differences between species were larger (*c.f*. Figures 2c and 4a). Most importantly, the relative infection rates differ between treatments. *Ulmerochorema rubiconum* had the highest infection rate in all treatments but the difference in infection rate between *U. rubiconum* and other species changed depending on the treatment. The relative mortality of *T. evansi* and *E. turbidum* also varied significantly with treatment as *E. turbidum* had a much lower infection probability in the Late spike treatment than *T. evansi* and this was reversed in the Low and Early spike treatments. This species by temperature interaction in infection probability means that in a fluctuating environment, there will be times when each species is more impacted by infection and others have a reprieve. While coexistence solely supported by these shifts would require each species to have conditions under which it is the least-infected, in reality many different processes are likely contributing to coexistence [44]. The shifts seen here in relative infection rates can contribute to stabilising coexistence by favouring different species in different conditions, even if they are not alone sufficient to maintain it.

Further differences between species could also alter a species’ ability to persist within a community. *Saprolegnia* spp. infection leads to mortality in caddisfly eggs [33] and, if the consequential decrease of hatchling numbers is not compensated for with lower mortality in later life stages [34, 43], it will lead to decreased population size and changes to relative abundances. As infection probability is much higher in *U. rubiconum*,* T. evansi* and *E. turbidum* than *U. seonum* in all treatments there is the potential for negative population consequences for those species and a shift in competitive relationships in larval life stages. Additionally, as *T. evansi* and *E. turbidum* lay many more eggs in a single egg mass than *U. rubiconum* [28], the number of hatchlings that survive from an infection which covers the same proportion of an egg mass would vary among these species. For example, if an egg mass was 50% infected and all other eggs hatched successfully, the number of hatchlings would be, on average, 586 for *E. turbidum*, 230 for *T. evansi* and only 97 for *U. rubiconum.* The egg numbers contribute to average fitness differences, where it appears *U. rubiconum* has an advantage based on abundance, despite its low eggs per egg mass. However, depending on larval density-dependence, this advantage is unlikely to scale linearly, providing an opportunity for the higher egg numbers in the other species.

Although infection probability is both temperature- and species-specific, the onset of infection was not affected by the same factors, suggesting the rate of infection spread caused the differences between groups rather than the initial infection timing. As infection onset was similar but total infection of eggs in treatments and species changed across groups, this suggests that a mechanism exists for avoiding infection or slowing the progress of infection throughout egg masses and to neighbouring egg masses. One possible mechanism driving varying infection spread rates could be species-specific oviposition preferences. For example, there is evidence that some Hydrobiosidae species prefer to oviposit on rocks that do not already have other egg masses present while other species congregate egg masses [31]. In this study, while rocks with egg masses were collected from a small number of riffles so that they were exposed to similar conditions for the first few hours after laying, we still saw large variations in the locations of egg masses on rocks and amount of congregating within and between species. Such preferences could lead to differences in infection due to the degree of hyphal spread able to occur between neighbouring egg masses. Another possible mechanism for decreased severity of infection could include an immune response within egg masses or individual eggs which are able to slow the progression of infections. Temperature can lead to a decreased immune response from heat stress [45] or increased pathogen replication and transmission [46] in other species, which could account for the result that constant high temperature had the greatest infection probability in this study, if applicable here. It is possible that different species have invested differently in defences against infection such as oviposition habits or immune response. Some may put more effort and resources into fighting the spread of infection, while others might invest in faster development of eggs to neonates in the hopes of hatching before infection takes hold.

In addition to differing infection probability across species, there were also differences in the variation of infection onset, as measured by CV, *c.f.* Figure 3b. Although the reason for this variation is unknown, one possible cause of changes in variation of day of infection could be that infection begins in eggs that are unviable and the timing is based on when the first egg within an egg mass dies. This infection mechanism occurs in fish eggs where infection by *Saprolegnia* spp. starts in unfertilised or unviable eggs and then quickly spreads to nearby healthy eggs [47]. If this opportunistic infection progression occurs in caddisfly egg masses, then the differences between species in the variation for timing of infection onset may be due to differences in the probability of eggs becoming compromised or unviable and therefore, more susceptible to infection.

Evidence is mounting that infection probability of *Saprolegnia* spp. is altered by another environmental factor in addition to temperature. Across three similar experiments ([34], results presented here and the single species in Appendix 2), total infection varied greatly and the impact of temperature varied depending on total infection rate. For example, in contrast to the current study, Taig *et al*. [34] found infection of *U. rubiconum* in the Early spike treatment to be significantly different from the Low treatment, however, the overall infection rate was also much different (36%; compared to 62% in the current study and 9% in the single species experiment). Thus, the effect of temperature across these three experiments was greatest at moderate overall infection rates. The effect of temperature is not simply due to the boundedness of infection, as each was analysed with logistic (beta-binomial) regression on the log-odds scale. Given that the experiment which had the greatest infection probability was the one which had additional infected egg solution added to each sample, the concentration of *Saprolegnia* spp. in the surrounding water, or perhaps even the number of hyphae specifically surrounding each rock, may alter baseline infection rates. The overall concentration of *Saprolegnia* spp. in the water is likely to be a result of a number of processes acting at larger spatial and temporal scales (e.g. other reservoir species or higher water temperatures over preceding weeks or months to promote growth). A similar outcome has been observed for fish species including carp (*Cyprinus carpio*), roach (*Rutilus rutilus*) and perch (*Perca fluviatilis*), where greater pathological changes were observed when exposed to increased levels of *Streptomyces griseus* spores under experimental conditions [48]. Although the cause of the changing rate of infection for caddisfly egg masses across experiments cannot be currently determined, our observations suggest that the relationship between temperature patterns and infection probability is likely interactive with other processes.

As this study was conducted under controlled laboratory conditions, it may not fully capture the complexity and variability of field environments. This is evidenced by the changed results between repeated experiments discussed above. Additionally, low sample sizes across some species also constrained some analyses. While low sample sizes limited statistical power for certain groups, the use of mixed-effects models and cautious interpretation helped minimise the risk of overstating these results. These limitations highlight the need for future studies that replicate these experiments under more natural field conditions and with greater sample sizes to better assess population-level consequences. Further, our study cannot directly demonstrate coexistence outcomes but can provide evidence of mechanisms which support coexistence (i.e. temperature and species specificity). To prove altered likelihood of coexistence, work is needed to establish whether these infection dynamics translate into long-term stabilising or equalising processes in natural systems.

Overall, we found that infection characteristics are complex and variable across species and temperature patterns. Our findings demonstrate that the host-parasite relationships between *Saprolegnia* spp. and the four caddisfly species in this study are both temperature- and species-specific. The species-specific response is suggestive of the host-predator interaction being a mechanism for maintaining coexistence through competitive dominance or frequency-dependent selection. Further, as species’ infection rates respond differently to temperature patterns, environmental fluctuations might provide the necessary separation of density-dependent feedbacks to promote coexistence by favouring different species in different conditions. Increased temperatures, as from climate change, may therefore yield overall directional shifts in community composition. Understanding these processes is essential for predicting how host-parasite interactions shape biodiversity in a changing climate by altering species densities and potentially ecosystem functioning if further interactions are interrupted.

## Supplementary Information

Below is the link to the electronic supplementary material.

Supplementary Material 1

## Data Availability

To foster transparency, our data and code is available on Figshare. 10.26187/deakin.28216244.

## Abbreviation

CV: Coefficient of variation
