# Supplementary material for: Temperature- and species-specific infection could modify stream insect communities
Source: Curr Microbiol. 2026 May 7;83(6):351. doi: 10.1007/s00284-026-04897-z (PMC13152880; doi:10.1007/s00284-026-04897-z)
Supplement: Supplementary file 1 — Supplementary Material 1 [file 284_2026_4897_MOESM1_ESM.docx]

**Temperature- and species-specific infection could modify stream insect communities**

Sarah A. Taig, Galen Holt, Georgia K. Dwyer and Rebecca E. Lester

# Appendix S1

# Section 1 – Sample collection methods

In early February 2023, when the weather was consistently warm with minimal wind and rain, eight riffles along the Steavenson and Taggerty rivers were chosen as experimental sites to collect naturally laid caddisfly egg masses. These sites had evidence of previous egg laying and were accessible from the road.

Rocks, collected from the bottom of the riverbed to ensure that they had no existing egg masses present, were scrubbed to remove any algae or debris and then set up to be emergent in the riffles in favourable oviposition locations. The rocks were large enough to not be washed away but small enough to fit in a 15-L experimental container with dimensions of 286 (W) x 403 (D) x 196 (H) mm. Each rock was marked with an oil pastel to be distinguishable from other existing emergent rocks. Depending on the size of the riffle, between 30 and 100 rocks were set up with newly positioned emergent rocks and left overnight for caddisfly laying.

To ensure that those newly laid egg masses became infected and infection characteristics could be measured, infected egg masses were collected to inoculate the samples in the laboratory the next day. The infected egg masses were scraped from rocks and stored in a 1-L bottle of river water and stored at 4ºC overnight.

The next morning, each of the rocks were inspected. Rocks that had one or more caddisfly egg masses present from the target species, which included *U. rubiconum, U. seonum, E. turbidum* and *T. evansi* were collected*.* The rocks were placed individually in numbered experimental containers, each filled with approximately 7 litres of water collected from Site 1 to control for the natural quantity of *Saprolegnia* spp. each egg mass would be exposed to from the river water. Temperature loggers were placed randomly into rock containers to measure temperature throughout the experiment. Additional water was also collected to top up any of the experimental samples if needed upon arrival at the laboratory or during the experiment. Rocks in experimental containers were transported to the Deakin University laboratory (Waurn Ponds, Victoria). Upon arrival, the rocks were again inspected and rocks with damaged egg masses were discarded; 43 rocks were included in the experiment.

# Section 2 – Randomisation of samples across chambers

To avoid individual chambers altering hatching or infection characteristics within the treatment groups, samples were randomised across appropriate chambers at the beginning and end of each spike treatment (e.g. Figure S1). Four chambers were used in total, with two held constant at each temperature and rocks moved between them for the spikes.


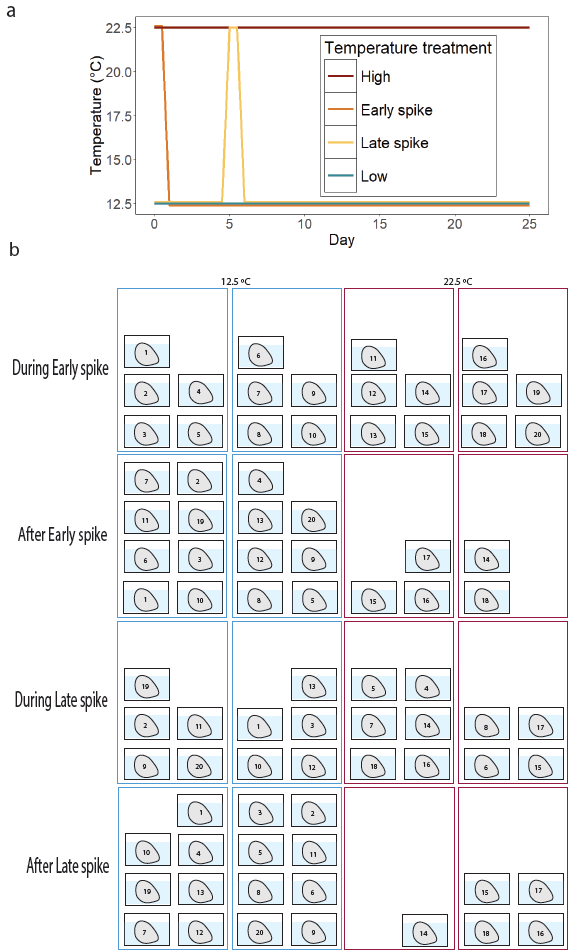


**Figure S1**: Example of the randomisation of rocks within chambers to prevent any impact of individual chambers (columns). Example treatment allocations for each rock are: Low = 1, 2, 3, 9, 10; Early spike = 11, 12, 13, 19, 20; Late spike = 4, 5, 6, 7, 8; High = 14, 15, 16, 17, 18.

# Section 3 – Single Species Experiment

To support the results for the treatment differences with greater sample size from a single caddisfly species another experiment was run with only *U. rubiconum* egg masses included. The methods for the single species study remained the same except for the following small changes to the sample collection and experimental set up.

*Method adjustments from main text*

The experiment was performed in early February 2022 and there were only five riffles from which the rocks with egg masses present were collected. The infected egg mass solution was not added to the samples as it was in the multi-species experiment.

The rocks collected had the target species *U. rubiconum.* We preferentially selected for rocks with multiple egg masses (rather than including rocks with single egg masses). Upon arrival at the lab, there were 49 rocks with egg masses included in the experiments due to one being damaged during transport. Random allocation of rocks to treatments and an odd number of total rocks samples resulted in uneven sample sizes across treatments (Low = 13, Early spike = 11, Late spike = 12, High = 13). Variation in the number of egg masses on each rock also led to an uneven number of egg masses in each treatment (Low = 68, Early spike = 54, Late spike = 60, High = 57), resulting in further differences among the treatments. Each egg mass was photographed within the first five days of the experiment and those photographs were used to count the number of individual eggs within each mass. These egg masses were monitored until every individual egg within an egg mass on a rock was infected or hatched which occurred in 11 to 23 days.

For the data analysis, the number of eggs in each egg mass for this single-species experiment were counted individually and were different for each egg mass. The same analyses were performed with only Treatment included as a predictor. Sample sizes for each of the analyses are presented in Table S1.

**Table S1:** The number of *U. rubiconum* egg masses or eggs included in each treatment group for the analysis of infection characteristics.

| **Treatment** | Total number of egg masses | Day of first infection (egg masses) | Coefficient of variation for day of first infection (egg masses) | Infection probability (eggs) | Coefficient of variation for maximum infection (egg masses) |
| --- | --- | --- | --- | --- | --- |
| **Low** | 69 | 2 | 2 | 12 056 | 69 |
| **Early Spike** | 54 | 1 | NA | 8538 | 54 |
| **Late Spike** | 62 | 7 | 7 | 10 382 | 62 |
| **High** | 57 | 11 | 11 | 8895 | 57 |
| **Total** | **242** | **21** | **23** | **39 887** | **242** |

*Results*

Q1. Does the day of infection vary with different temperature patterns?

Significant differences between treatments were not supported by the analysis of deviance (Table S2). The analysis of deviance reflected the results of the main multi-species analyses.

When comparing individual treatments the High treatment had significantly earlier day of infection than the Low treatment, but no other treatment differences were observed (Table S3, Figure S2).

**Table S2:** Analysis of deviance Table (Type II Wald chi squared tests) for the first day of infection.

|  | Chi squared | Degrees of freedom | **p-value** |
| --- | --- | --- | --- |
| Treatment | 5.411 | 3 | 0.144 |

**Table S3:** Comparisons to the Low treatment from a Poisson regression for treatment differences in the day of infection. Standard error is abbreviated to SE. n = 21 egg masses.

| **Contrast to Low *U. rubiconum*** | **Estimate** | **SE** | **t-value** | | **~ p-value** |
| --- | --- | --- | --- | --- | --- |
| (intercept) | 2.327 | 0.406 | **5.737** | | **<0.0001** |
| Early spike | -1.294 | 0.849 | -1.524 | | 0.128 |
| Late spike | -0.405 | 0.516 | -0.786 | | 0.432 |
| High | -0.955 | 0.472 | **-2.021** | | **0.043** |
|  | | | | | |
| **Random effects** | **Variance** | **Standard deviation** | |  |  |
| Cluster: Rock | 3.511e-9 | 5.925e-5 | |  |  |
| Rock | 0.218 | 0.467 | |  |  |


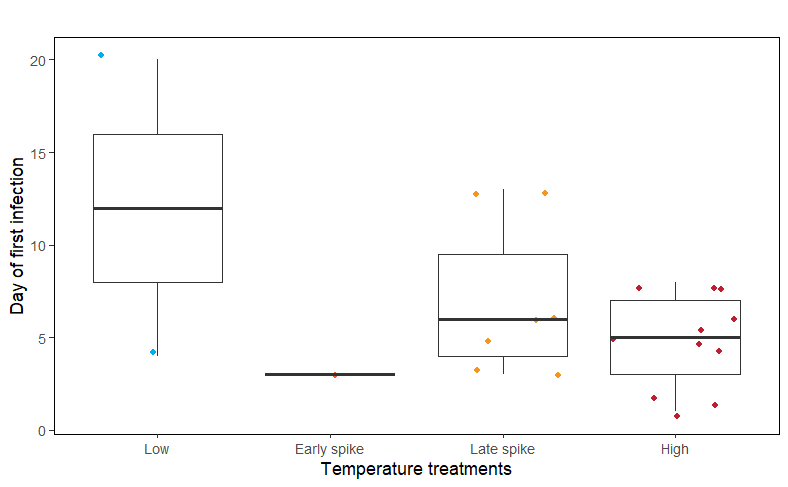


**Figure S2:** The day of infection for each treatment. The coloured points represent egg masses. There was no significant differences for the treatment term. The bold line within the boxplots indicates the median for each grouping. The boxes represent the interquartile ranges. Whiskers extend to the smallest and largest values within 1.5 times the interquartile ranges. In total 21 egg masses were included in these analyses.

There was also no difference in the coefficients of variation for the day of first infection between any of the treatments (Figure S3). The Early spike is not included in this analysis due to low infection rates. The results did not match the results of the multi-species experiment where the coefficient of variation of *U. rubiconum* was smaller in the Late spike treatments than the other treatments.


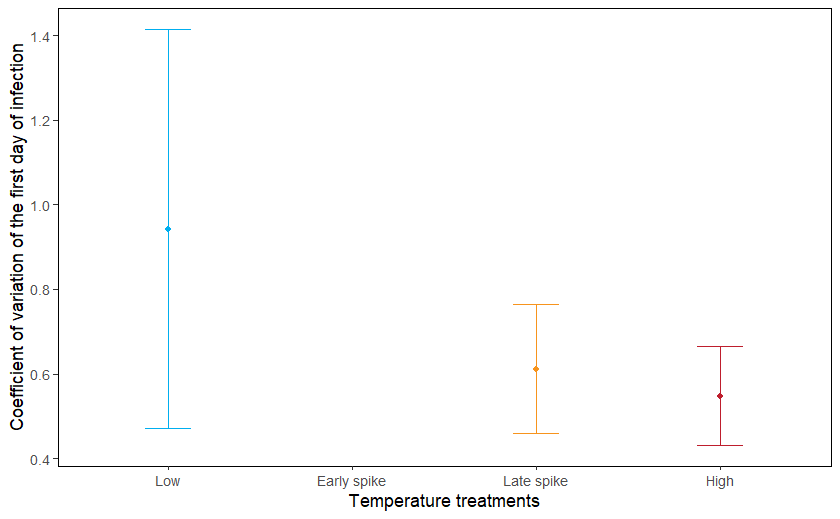


**Figure S3:** The coefficient of variation for the day of infection differences between treatments. Coloured points represent the coefficient of variation for each species and error bars are the standard error. In total 21 egg masses were included in this analysis. Early spike is not present due to low infection rates.

Q2. Does the probability of infection, and therefore mortality, vary with different temperature patterns?

There were significant differences between treatments with the High treatment having the highest infection probability (Figure S4; Table S4; Table S5). This supported the results of the multi-species experiment where the Treatment term was also significant.

**Table S4:** Analysis of Deviance Table (Type I Wald chi squared tests) for the probability of infection.

|  | Chi squared | Degrees of freedom | **p-value** |
| --- | --- | --- | --- |
| Treatment | 9.059 | 3 | **0.029** |

**Table S5:** Comparisons to *U. rubiconum* from the Low treatment from beta binomial logistic regressions for treatment and species differences in the probability of infection. Standard error is abbreviated to SE. n = 71 710 eggs.

| **Contrast to Low *U. rubiconum*** | **Estimate** | **SE** | **t-value** | **~ p-value** |
| --- | --- | --- | --- | --- |
| (intercept) | -5.897 | 0.694 | **8.488** | **<0.0001** |
| Early spike | -0.183 | 1.045 | 0.175 | 0.860 |
| Late spike | 0.061 | 0.970 | 0.063 | 0.950 |
| High | 2.199 | 0.929 | **2.366** | **0.018** |
| Egg mass : Cluster: Rock | -0.144 | 0.187 | NA | NA |
| Cluster: Rock | -0.311 | 0.232 | NA | NA |
| Rock | -1.478 | 0.553 | NA | NA |


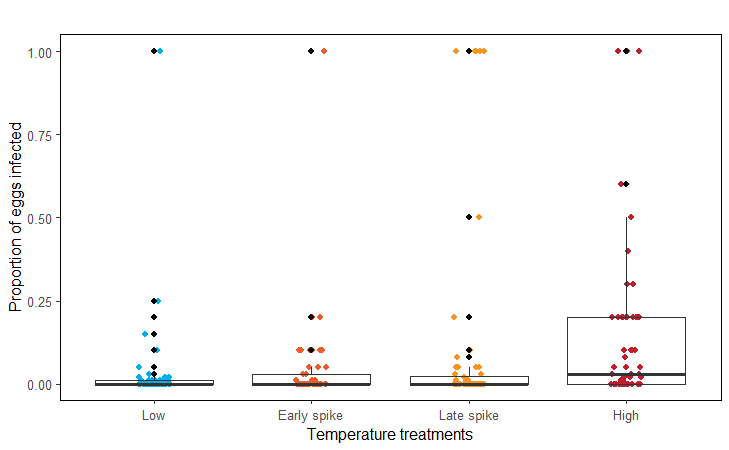


**Figure S4:** The proportion of eggs infected with *Saprolegnia* spp. across treatments. The bold line within the boxplots indicates the median for each species. The larger boxes represent the interquartile ranges. Whiskers extend to the smallest and largest values within 1.5 times the interquartile ranges and black points correspond to outlying egg masses. Each coloured point represents one egg mass. Eggs (39 887) from all the egg masses (242) were included in this analysis.

The coefficient of variation decreased with increased temperatures (Figure S5). This differs from the overall trend seen in the multi-species experiment where all the treatments had similar average coefficients of variation. It also does not match the trend for *U. rubiconum* in that experiment, which had lower coefficients of variation in the Low and High treatments compared to the spike treatments.


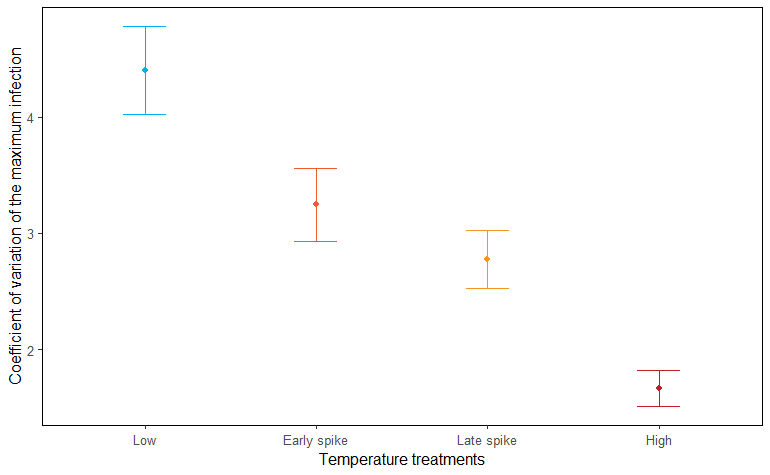


**Figure S5:** The coefficient of variation for the maximum infection differences between treatments. Coloured points represent the coefficient of variation and error bars are the standard error. In total 242 egg masses were included in these analyses.

# Section 4 – Complete Poisson regression for first day of infection (Q1)

To determine any treatment and species differences in the first day of infection a model including both terms and their interaction was run. An analysis of deviance of this model was performed to test significance of the terms and presented in the main results (Table 2a). The full model results with the estimates for each level is given in (Table S5).

**Table S5:** Comparisons to *U. rubiconum* from the Low treatment from a Poisson regression for treatment and species differences in the first day of infection.

| **Contrast to Low *U. rubiconum*** | **Estimate** | **SE** | **t-value** | **~ p-value** | |
| --- | --- | --- | --- | --- | --- |
| (intercept) | 1.668 | 0.103 | **16.157** | **<0.001** | |
| Early spike | 0.041 | 0.133 | 0.309 | 0.757 | |
| Late spike | -0.017 | 0.147 | -0.117 | 0.907 | |
| High | -0.230 | 0.141 | -1.626 | 0.104 | |
| *T. evansi* | 0.034 | 0.323 | 0.106 | 0.915 | |
| *E. turbidum* | -0.025 | 0.244 | -0.104 | 0.917 | |
| Early spike : *T. evansi* | -0.461 | 0.450 | -1.025 | 0.305 | |
| Late spike : *T. evansi* | -0.129 | 0.414 | -0.311 | 0.756 | |
| High : *T. evansi* | -0.310 | 0.417 | -0.744 | 0.457 | |
| Early spike : *E. turbidum* | -0.486 | 0.411 | -1.183 | 0.237 | |
| Late spike : *E. turbidum* | -0.018 | 0.525 | -0.035 | 0.972 | |
| High : *E. turbidum* | 0.016 | 0.324 | 0.049 | 0.961 | |
| **Random effects** | | | | | |
|  | **Variance** | **Standard deviation** | | | |
| Cluster: Rock | **<0.0001** | **<0.0001** | | | |
| Rock | 0.006 | 0.074 | | |  |

Standard error is abbreviated to SE. n = 148 egg masses.

# Section 5 – Results of the time to infection analyses (Q1) without the interaction term

As the interaction term was not significant in the time to infection analysis (Table 2a), the analysis was repeated without the interaction. There were no significant differences in the time to first infection in the remaining terms, Species and Treatment, which supports the results presented in the main text (analysis of deviance, Table S6 and full estimates, Table S7).

**Table S6:** Analysis of deviance table (Type II Wald chi squared tests) for the first day of infection.

|  | Chi squared | Degrees of freedom | **p-value** |
| --- | --- | --- | --- |
| Treatment | 4.871 | 3 | 0.182 |
| Species | 2.523 | 2 | 0.283 |

**Table S7:** Comparisons to *U. rubiconum* from the Low treatment from a Poisson regression for treatment and species differences in the first day of infection. Confidence interval is abbreviated to CI, Standard error to SE. n = 148 egg masses.

| **Contrast to Low *U. rubiconum*** | **Estimate** | **SE** | **t-value** | | **~ p-value** |
| --- | --- | --- | --- | --- | --- |
| (intercept) | 1.701 | 0.096 | **17.782** | | **<0.0001** |
| Early spike | -0.036 | 0.125 | -0.287 | | 0.774 |
| Late spike | -0.032 | 0.133 | -0.240 | | 0.810 |
| High | -0.248 | 0.126 | -1.959 | | 0.050 |
| *T. evansi* | -0.204 | -0.143 | -1.424 | | 0.154 |
| *E. turbidum* | -0.119 | 0.138 | -0.861 | | 0.389 |
|  | | | | | |
| **Random effects** | **Variance** | **Standard deviation** | |  |  |
| Cluster: Rock | 1.894e-9 | 4.352e-5 | |  |  |
| Rock | 6.413e-3 | 8.008e-2 | |  |  |

# Section 6 – Analysis of Deviance results for infection probability (Q2) with differed term orders

As the order of the terms can significantly change the results of Type I analysis of deviance, the terms were reordered and the analysis rerun. The Species and Species by Treatment interaction terms were significant, but the Treatment term was not in this sequence (Table S8), which differed from the main results. This is likely due to multicollinearity between the Species and Treatment terms; with the Species term being highly significant, most of the variance explained by Treatment is already accounted for.

**Table S8:** Analysis of deviance table (Type I Wald chi square tests) for the probability of infection where the order of the terms were reordered.

|  | Chi squared | Degrees of freedom | **p-value** |
| --- | --- | --- | --- |
| Species | 79.221 | 3 | **<0.0001** |
| Treatment | 4.485 | 3 | 0.214 |
| Species:Treatment | 19.915 | 9 | **0.019** |

# Section 7 – Complete beta binomial (logistic) regression for infection probability

To determine any treatment and species differences in the probability of infection a model including both terms and their interaction was run. An analysis of deviance of this model was performed and presented in the main results to identify significant model terms (Table 2). The full model results with the estimates for each level is given in (Table S9).

**Table S9:** Comparisons to *U. rubiconum* from the Low treatment from beta binomial logistic regressions for treatment and species differences in the probability of infection.

| Contrast to Low *U. rubiconum* | Estimate | SE | t-value | ~ p-value |
| --- | --- | --- | --- | --- |
| (intercept) | 8.318 | 3.006 | **2.767** | **0.005** |
| Early spike | -4.734 | 3.536 | -1.339 | 0.181 |
| Late spike | 0.992 | 3.735 | 0.265 | 0.791 |
| High | -3.402 | 3.490 | -0.975 | 0.330 |
| *U. seonum* | -11.206 | 4.298 | **-2.608** | **0.009** |
| *T. evansi* | -4.734 | 3.595 | -1.317 | 0.188 |
| *E. turbidum* | -4.811 | 3.465 | -1.388 | 0.165 |
| Early spike : *U. seonum* | -2.871 | 4.441 | -0.646 | 0.518 |
| Late spike : *U. seonum* | -0.447 | 5.699 | -0.078 | 0.938 |
| High : *U. seonum* | 8.520 | 5.745 | 1.483 | 0.138 |
| Early spike : *T. evansi* | 1.902 | 4.032 | 0.472 | 0.637 |
| Late spike : *T. evansi* | -10.202 | 4.730 | **-2.127** | **0.031** |
| High : *T. evansi* | 6.801 | 4.070 | 1.671 | 0.095 |
| Early spike : *E. turbidum* | 2.196 | 3.829 | 0.574 | 0.566 |
| Late spike : *E. turbidum* | -4.475 | 3.979 | -1.125 | 0.261 |
| High : *E. turbidum* | 10.144 | 4.276 | **2.372** | **0.017** |
| Egg mass : Cluster: Rock | -0.427 | 0.188 | NA | NA |
| Cluster: Rock | 0.043 | 0.611 | NA | NA |
| Rock | 0.380 | 0.324 | NA | NA |

Standard error is abbreviated to SE. n = 71 710 eggs.

# Section 8 – Order of treatments for infection proportion

Specific inequalities can be seen in the order of which treatments increase in infection proportion (Table S10).

**Table S10:** The ranked order of treatments from the largest proportion of eggs infected within a treatment to the smallest proportion for each species.

| Species | Treatment rank | | | |
| --- | --- | --- | --- | --- |
|  | 1 | 2 | 3 | 4 |
| *U.* *rubiconum* | High | Low | NA | Late spike and Early spike |
| *U. seonum* | High | Late spike | Low | Early spike |
| *E. turbidum* | High | Low | Early spike | Late spike |
| *T. evansi* | High | Late spike | Low | Early spike |
